# Protein Supplementation Does Not Further Increase Latissimus Dorsi Muscle Fiber Hypertrophy after Eight Weeks of Resistance Training in Novice Subjects, but Partially Counteracts the Fast-to-Slow Muscle Fiber Transition

**DOI:** 10.3390/nu8060331

**Published:** 2016-06-01

**Authors:** Antonio Paoli, Quirico F. Pacelli, Pasqua Cancellara, Luana Toniolo, Tatiana Moro, Marta Canato, Danilo Miotti, Marco Neri, Aldo Morra, Marco Quadrelli, Carlo Reggiani

**Affiliations:** 1 Department of Biomedical Sciences, University of Padova, Padova 35131, Italy; francesco.pacelli@unipd.it (Q.F.P.); linacancellara@gmail.com (P.C.); luana.toniolo@unipd.it (L.T.); tatiana.moro.phd@gmail.com (T.M.); marta.canato@unipd.it (M.C.); carlo.reggiani@unipd.it (C.R.); 2 Salvatore Maugeri Foundation, Pavia 27100, Italy; danilo.miotti@fsm.it; 3 AIFeM (Italian Medicine and Fitness Federation), Ravenna 48121, Italy; neri@cervia.com; 4 Euganea Medica, Diagnostic Centre, Via Colombo 13, Albignasego (Padova) 35020, Italy; aldo.morra@euganeamedica.it (A.M.); marco.quadrelli@euganeamedica.it (M.Q.)

**Keywords:** nutrition, supplementation, whey protein, myosin isoform, strength training, single muscle fiber mechanics

## Abstract

The response to resistance training and protein supplementation in the latissimus dorsi muscle (LDM) has never been investigated. We investigated the effects of resistance training (RT) and protein supplementation on muscle mass, strength, and fiber characteristics of the LDM. Eighteen healthy young subjects were randomly assigned to a progressive eight-week RT program with a normal protein diet (NP) or high protein diet (HP) (NP 0.85 *vs.* HP 1.8 g of protein·kg^−1^·day^−1^). One repetition maximum tests, magnetic resonance imaging for cross-sectional muscle area (CSA), body composition, and single muscle fibers mechanical and phenotype characteristics were measured. RT induced a significant gain in strength (+17%, *p* < 0.0001), whole muscle CSA (*p* = 0.024), and single muscle fibers CSA (*p* < 0.05) of LDM in all subjects. Fiber isometric force increased in proportion to CSA (+22%, *p* < 0.005) and thus no change in specific tension occurred. A significant transition from 2X to 2A myosin expression was induced by training. The protein supplementation showed no significant effects on all measured outcomes except for a smaller reduction of 2X myosin expression. Our results suggest that in LDM protein supplementation does not further enhance RT-induced muscle fiber hypertrophy nor influence mechanic muscle fiber characteristics but partially counteracts the fast-to-slow fiber shift.

## 1. Introduction

It is well known that resistance training (RT) is the most effective stimulus to promote muscle hypertrophy. Since the early 1980s, many studies have investigated at a cellular and molecular level the effect of RT on skeletal muscle [1,2,3,4] enlargement. Although the underlying precise mechanism is still elusive [5], the efficacy of RT in inducing muscle fiber hypertrophy, especially of the type 2 fibers, is well documented in the literature [6]. Exercise, however, is only one side of the coin: the other side is the amount of daily protein intake required for muscle hypertrophy. Muscle responses to RT under various protein intake regimens have recently generated considerable research interest [7,8,9,10,11,12]. It has been demonstrated that RT is capable of enhancing the synthesis of muscle proteins (including myofibrillar proteins) and that augmented protein feeding potentiates its effects [7,13,14]. It is commonly accepted that a high protein diet is necessary to achieve muscle hypertrophy but how RT and high protein diets interact to induce muscle hypertrophy is not clear, probably due to some limits in research studies, as stated by Cermack and colleague in a recent meta-analysis [15].

It is important to point out that the majority of studies on muscle hypertrophy and protein supplementation was done on lower limbs and, more specifically, on quadriceps’s vastus lateralis. Upper limb muscles have been less studied, in spite their significant involvement in RT protocols. In addition, being free of day-long postural duties, upper limbs can be considered more suited for a study aimed at identifying the direct effects of RT as well as protein intake. In addition, the composition of the upper limbs shows a predominance of fast fibers, confirming their restricted activity to a specific period of the day [16,17]. Among upper limb muscles, no study has been performed until now on the response of the latissimus dorsi muscle (LDM) to strength training and protein supplementation, despite its relevance for a number of functions and exercises (throwing, climbing, but also locomotion in water, microgravity, or in a wheelchair). The main question is how upper limb muscles and, more specifically, LDM respond to RT and protein supplementation. To answer this question we sought to study the changes in LDM muscle fibers after eight weeks of RT with two different daily protein intakes in 18 healthy volunteers.

## 2. Subjects and Methods

### 2.1. Subjects

Eighteen undergraduate male students (age 24.9 ± 5.3 years; 79.5 ± 10.5 kg body weight, 182 ± 7.8 cm height) enrolled in a “Human Movement Science” course at the University of Padova responded to an invitation to participate to the study. Respondents provided written informed consent to take part in the study and were screened for the presence of diseases or conditions that would place them at risk for adverse responses to exercise. All participants were healthy, non-obese non-smokers and were not taking any medications. None of them had been engaged in resistance training on a regular basis for at least two years before the start of the study and a few subjects had only minimal previous RT experience (2–3 months). All participants were active for 5–6 h/week in various team sports (soccer, volleyball, basketball). Subjects were first matched in pairs based on age and level of physical activity. The study was approved by the Ethical Commission of the Salvatore Maugeri Foundation (Pavia, Italy) where biopsy and medical tests were performed, in accordance with the Helsinki declaration of 1995 as modified in 2000.

### 2.2. Study Design

**Figure 1:**
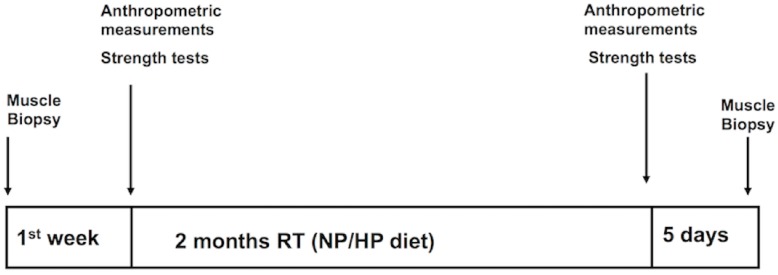
Experimental design. LDM = latissimus dorsi muscle; RT = resistance training; NP = normal protein diet; HP = high protein diet.

During the first week after recruitment, all participants underwent physical and physiological tests and muscle biopsy samples were collected. Subjects were randomly allocated to the high protein group (HP) or to the normal protein group (NP). During the training period HP group consumed 1.8 g·kg^−1^·day^−1^ of protein while the NP group consumed 0.85 g·kg^−1^·day^−1^ of protein. All participants followed the same resistance training program for eight weeks. At the end of the training period the same sequence of tests was repeated and biopsy sampling was performed. A scheme of the study design is shown in Figure 1.

### 2.3. Resistance Training Protocol

Supervised training sessions were performed in two non-consecutive days/week for the first two weeks and in three non-consecutive days/week for the following six weeks under qualified exercise trainer supervision and, in particular, during the first two weeks all exercises were supervised to ensure correct lifting technique. The exercises performed throughout the program were: bench press, latissimus pulldowns, seated rows, shoulder press, biceps hammer curls, and dumbbell lying external rotation. During the first week of training the participants performed two sets of 9–11 repetitions at 75%–80% 1 RM with 2-min breaks between each set in all exercises except for hammer curl (1.5 min) and dumbbell lying external rotation (1 min). Thereafter, from the second to the fourth week the training volume was elevated to three sets. In the fifth week the intensity of training was elevated to 80%–85% 1 RM with three sets of 6–8 repetitions. The recovery between each set was 3 min for all exercises except for the hammer curl (2 min) and dumbbell lying external rotation (1.5 min). From the sixth to the eighth week the training volume was elevated to four sets. The participants were instructed to perform the repetitions rapidly (1 s) during the concentric phase and then return the load through the eccentric (lowering) phase at a slower and more controlled speed (1.5/2 s) [18]. The load was adjusted every week according to the actual number of repetitions performed. A general rule of a 5% adjustment for every two repetitions of deviation (increase) from the desired number of repetitions was used or, more generally, weights were adjusted to ensure that subjects were unable to perform more than one repetition beyond those scheduled [19]. Exercise compliance was monitored using a daily exercise schedule completed daily by the trainers and returned to the research staff every week.

### 2.4. Nutritional Assessment

Before assigning the HP diet or NP, during a preliminary interview (aimed at excluding a previous high protein diet), all participants filled in a record of the previous 24 h as the first page of the seven-day diary of foods eaten. The participants were informed how to record food intake. The diaries were collected every week in the course of the study, in order to check the food intake. Moreover a prescriptive, fixed-menu plan was given to all participants together with explanations of the different kinds of diets. For all diets the total daily caloric intake was divided into five meals. The individual daily caloric needs ware calculated according to body composition and adjusted for daily activity [20,21]. The diet macronutrient percentage distribution (see Table 1) was as follows: for the HP group proteins were 28% of TDC (total daily calories), fats were 25%, and carbohydrates represented 47%; for NP the percentages were 13%, 25%, and 62% of the TDC for proteins, fats, and carbohydrates, respectively. Both the NP and HP diets were isocaloric regarding the daily energy needs of subjects, while the surplus of calories given by protein supplements was substituted by carbohydrates in the diet. Protein intake in the NP group was 0.85 grams per kg of body weight while in the HP group it was 1.8 g per kg of body weight. A whey protein supplement (PD Whey 100 GensanSrl, Ospedaletto (PI), Italy) was given to HP subjects to allow them to reach the desired quantity of protein (1.8 g per kg of body weight) while a placebo (water with no caloric sweetener) was given with the same modalities to NP subjects. During the warm-up (10 to 15 min before the beginning of each training session) and 1 h after the end of the training session, the subjects received 250 mL of a beverage containing 15–20 grams of protein, for a daily amount of 30–40 grams or a placebo. The amino acid composition of the protein supplement was as follows (for 10 grams): leucine (1.12 g), isoleucine (0.46 g), valine (0.43 g), methionine (0.2 g), lysine (0.9 g), threonine (0.41 g), phenylalanine (0.3 g), alanine (0.47 g), arginine (0.2 g), aspartic acid (0.95 g), cysteine (0.28 g), glutamic acid (1.37 g), glycine (0.14 g), proline (0.4 g), serine (0.28 g), tyrosine (0.32 g), histidine (0.16 g), and tryptophan (0.3 g). At the end (week 8) of the exercise intervention, the subjects recorded three days of weighted dietary records (Thursday–Saturday) to assess potential changes in daily food intake that might have occurred during the intervention period. Food intake records were scrutinized by a nutritionist and analyzed with DietNext (GSA-Tea Srl, Caldogno (VI), Italy). Dietary intake was calculated for the entire day. The placebo contained a non-caloric sweetener and colorant. The subjects in both the protein and placebo group did not eat anything 60 min before and 30 min after each RT session (with the exception of supplements or the placebo).

### 2.5. Exercise Testing

Subjects were tested for their one repetition maximum (1 RM) on six different exercises after a short general (10 min on a stationary bike) warm-up and a specific warm-up consisting of 15 repetitions at an estimated 30% of maximum. The 1 RM testing procedure is described elsewhere [22]. The six tested exercises that involved the recruitment of upper limb muscles were: bench press, latissimus pull down, seated row, shoulder press, biceps hammer curl, and dumbbell lying external rotation.

### 2.6. Anthropometric Measurements

Muscle and fat amounts and percentages were assessed by skinfold measurements, which are highly related to percentage body fat in fit and healthy young individuals [23,24,25]. We used a software (Fitnext^®^, Caldogno, Vicenza, Italy) that includes nine skinfolds (triceps, biceps, pectoral, subarmpit, subscapular, iliac crest, mid-abdominal, anterior thigh, medial calf), six bone circumferences (arm, forearm, waist, hip, thigh, calf), four bone diameters (elbow, wrist, knee, ankle), waistline, and hip circumference measurement [26]. Anthropometric measurements were performed according to the Anthropometric Standardization Reference Manual [25]. Weight was measured to the nearest 0.01 kg using an electronic scale (Tanita BWB-800 Medical Scales, Tanita Co. Arlington Heights, IL, USA), and height to the nearest 1 cm using a Harpenden portable stadiometer (Holtain Ltd., Pembrokeshire, UK). Skinfolds were measured to the nearest 1 mm using a Holtain caliper (Holtain Ltd.), and circumferences to the nearest 0.001 m using an anthropometric tape. All measurements were taken by the same operator (PQF) before and during the study according to standard procedures [27,28]. Muscle area of the dominant arm (that is synergic with LD in the analyzed tasks) was assessed by anthropometric measurements and the measurement was validated with magnetic resonance (MR) measurements. MR images were acquired using a 1.0 Tesla Scanner, Philips Panorama (Philips Medical System, Best, The Netherlands), using a dedicated solenoid coil, obtained with axial T1 weighted images (field of view 150 mm, TR 500 ms, TE 20 ms, 2 NSA), orientated along the muscle long axis. All analyses were conducted by an investigator blinded with regard to each subject’s protocol. The CSA calculations were performed three times (mean ICC = 0.99) and the mean value was used as a single data point for statistical analysis.

### 2.7. Muscle Biopsy and Muscle Fiber Analysis

Biopsy samples were collected from the lateral edge of the LDM at the level of the 5th rib. Tru-cut needles (PRECISA 1410 HS Hospital Service S.p.A, Latina, Italy) with a diameter of 14 G and an insertion cannula length of 100 mm were used. To determine the spatial coordinates of LDM fibers a strict palpation anatomy protocol was adopted. After identifying the biopsy location, the subjects were asked to lie down on their side and, after local anesthesia with 2 cc of xylocaine 2% and sterilization with Betadine, the needle was inserted into the muscle. The protocol of fine needle biopsy in LDM is described in detail elsewhere [29]. In each subject, three samples were collected in subsequent insertions of the inner notched rod of the needle. The average yield from each sampling was 4 mg, which corresponds to a cylinder of approximately 0.7–0.9 mm diameter, cross-sectional area of approximately 0.5 mm^2^, and length of about 8 mm (*i.e.*, the full length of the notch on the inner rod). Approximately 500 fiber segments were present in each sample and among these approximately 100 segments reached a length above 0.5 mm, thus allowing their use in single fiber mechanical experiments. The sample to be used for this purpose was immediately immersed in high potassium, high EGTA solution, usually indicated as a skinning solution (see below), mixed in equal parts with glycerol. Once immersed in this solution, the sample could be stored at −20 °C for up to two weeks.

On the day of the experiment, the samples were washed with skinning solution with ATP, and single fibers were manually dissected under a stereomicroscope and permeabilized with 1% Triton X-100. Fiber segments with an average length of 0.7 mm were mounted in the set-up between the force transducer (model AME-801; SensorOne, Sausalito, CA, USA) and the motor (SI, Heidelberg, Germany) equipped with a displacement transducer by means of light aluminum clips. The fiber segment was immersed in a drop of relaxing solution and, after measuring length, diameter, and sarcomere length at 400× magnification, were stretched by 20%. Cross-Sectional Area (CSA) was calculated from the measurements of three diameters (at 400× magnification), assuming the cylindrical shape of the fiber. The fiber was then transferred into the preactivating solution and finally maximally activated by immersion in the activating solution (pCa 4.6) at 12 °C. Isometric force (Fo) was measured in four subsequent maximal activations and the average value was calculated. The fiber segment was then removed from the setup and stored in Laemmli solution for electrophoretic determination of MyHC isoform composition.

Skinning, relaxing, preactivating, and activating solutions were prepared as previously described [30]. Their millimolar composition was as follows: (1) skinning solution contained 150 potassium propionate, 5 magnesium acetate, 5 ATP, 5 EGTA, and 5 KH_2_PO_4_; (2) relaxing solution contained 100 KCl, 20 imidazole, 5 MgCl_2_, 5 ATP, and 5 EGTA. The preactivating solution was similar to the relaxing solution except that the EGTA concentration was reduced to 0.5 mM and 25 mM creatine phosphate and 300 U/mL creatine phosphokinase were added, whereas the activating solution was similar to the relaxing solution with the addition of 5 mM CaCl_2_, 25 mM creatine phosphate, and 300 U/mL creatine phosphokinase. The pH of all solutions was adjusted to 7.0 at the temperature at which solutions were used (12 °C). Protease inhibitors (10 μM E-64 and 40 μM leupeptin) were present in all solutions.

Each fiber was classified by its MyHC isoform composition and characterized by its cross-sectional area (CSA), isometric force (Fo), and isometric tension (Po), obtained by normalizing Fo to CSA. To ensure that the response of each individual was equally represented, the fibers from a given subject were pooled to calculate an individual weighted mean [31].

Proteins for gel electrophoresis were prepared from the remaining sample after single fiber dissection and from a sample specifically collected for this purpose. The tissue samples were solubilized in Laemmli solution (62.5 mMTris, pH 6.8, 10% glycerol, 2.3% SDS, 5% β-mercaptoethanol, with 0.1% E-64 and 0.1% leupeptin as anti-proteolytic factors). After heating for 5 min at 80 °C, appropriate amounts of the protein suspension were loaded onto polyacrylamide gels (about 1 µg of total protein/lane). For single fiber identification, the whole fiber segment was solubilized in 10 µL of Laemmli solution, and 2–3 µL were loaded onto gels. The separation of MyHC isoforms was achieved on 8% polyacrylamide slab gels with a protocol derived from Talmadge and Roy [32] with some modifications. Slabs 18 cm wide, 16 cm high, and 1 mm thick were used. Electrophoresis was run at 4 °C for 24 h, at 70 V for 1 h and 230 V for the remaining time. Three bands were separated in the region of 200 kDa, corresponding (in order of migration from the fastest to the slowest) to MyHC-1, MyHC-2A, and MyHC-2X. Gels for single fiber classification were silver stained (Bio-Rad Silver stain plus), while gels for MyHC isoform distribution in multi-fiber samples were stained with Coomassie Blue to allow a quantitative determination of the relative proportions of the three MyHC isoforms. The relative proportions of MyHC isoforms were determined by the measurement of the brightness-area product (BAP) (*i.e.*, the product of the area of the band by the average brightness, subtracted local background after black-white inversion) with the accuracy of 600 dpi.

### 2.8. Immunofluorescence

In order to determine the nuclear density and the nuclear domain size, from the fiber bundles immersed in skinning solution (see above) single muscle fibers were manually dissected and fixed with 4% paraformaldehyde in PBS for 20 min at room temperature. The fibers were permeabilized with 0.1% Triton X-100 in PBS at room temperature, and then incubated in 10% normal goat serum for at least 30 min to block aspecific antibody binding. Mouse anti α-actinin (clone EA-53 Sigma Aldrich St Louis, Mo, USA), a monoclonal antibody, was applied (1:2000) at room temperature in PBS. After three washes (10 min each), fluorescent secondary Alexa-568 anti-mouse, (Molecular Probes) antibody was incubated for 2 h at room temperature. For visualization of nuclei, single fibers were stained with Hoechst (25 μg/mL; Sigma-Aldrich) for 10 min. After a final wash in 0.1 M PB, the fibers were mounted in 100% glycerol (Sigma-Aldrich) and covered with a coverslip. The fibers were viewed with a confocal microscope (VICO, Nikon, Firenze, Italy). Serial confocal optical sections (step size: 0.5 µm) were collected by scanning the fiber in only one direction (from top to bottom). The fiber segment volume was reconstructed by adding the volume of the individual sections, each obtained as the product of thickness section (*z* axis) by surface area (*xy* axis). The sections were then collapsed on the *z* axis and the number of nuclei was counted. From nuclei number and volume, the nuclear density (nuclei/106 μm^3^) and the nuclear domain size (μm^3^/nucleus) were obtained. It is worthwhile to observe that the combination of fixation and compression between slides leads to values comparable to those obtained with similar procedures [33], but lower than those obtained with skinned unfixed fibers [34].

### 2.9. Statistical Analysis

Baseline differences between the training and control groups for the reported variables were tested using independent sample Student’s *t*-tests. The normality of the data was checked and subsequently confirmed using the Kolmogorov–Smirnov test. A repeated measurement two-way ANOVA, using the data as a mixed factorial design (according to the software’s setting) with one between-subjects factor (treatment/diet) and one within-subjects factor (pre-test/post-test) was used and a Sidak’s multiple *post hoc* comparison was used when appropriate. Fibers were individually pooled and average values of fiber CSA and mechanical characteristics (Fo and Po) were calculated as weighted averages (*i.e.*, the individual average value of each class of fiber was weighted to account for possible differences in the numbers of fiber sampled and analyzed in each subject). When the HP and NP data were merged and analyzed a paired Student’s *t*-test was used. The present sample size was selected based on a power analysis; the sample size analysis using a power of 0.8 and an α-level of 0.05 indicated that the minimum number of subjects required to detect a significant difference when pools of isolated fibers are considered (*i.e.*, a within-subject variability of 30%) was eight.

All data were analyzed by using Prism5 GraphPad software (Abacus Concepts GraphPad Software, San Diego, CA, USA). Data are expressed as means and standard errors, unless otherwise indicated. Significance was settled at *p* < 0.05.

## 3. Results

No significant differences were detected between the training and control groups for the reported variables at the baseline.

### 3.1. Diet and Training Compliance

The analysis of diaries of foods eaten showed good compliance to the fixed menu for both groups. Examples of diet composition are given in Table 1. Each subject respected the prescribed daily caloric intake (±3%) and the prescribed protein intake (HP ± 3.7% and NP ± 4.1%). Subjects performed all scheduled training sessions.

### 3.2. Muscle Strength and Body Composition

**Figure 2:**
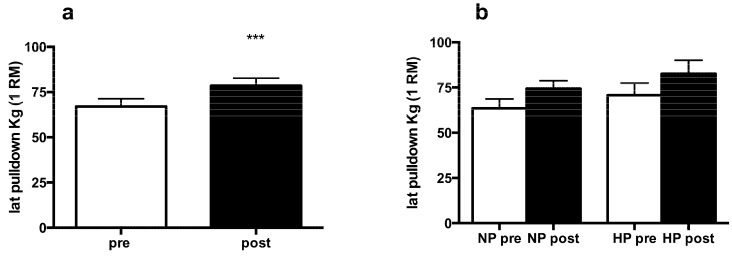
Effects of training and protein amount in diet on 1 RM at latissimus pull down. (**a**) All subjects (*n* = 18); (**b**) subjects were divided into high protein (HP, *n* = 9) and normal protein groups (NP, *n* = 9). A paired Student’s *t*-test (merged data) and a mixed model ANOVA (with one between-subjects factor and one within-subjects factor) were used. MEANS and Standard Errors. *** *p* < 0.001.

The resistance training protocol induced a significant increase in 1 RM at latissimus pulldown (from 670.8 ± 170 N to 785 ± 182 N; *p* < 0.0001) but without any significant differences between the HP and NP groups (Figure 2). Muscle area of the upper limb increased significantly (*p* = 0.024) from 45.13 ± 3.3 cm^2^ to 47.94 ± 4.4 cm^2^, as shown by MR (Table 2), without any significant difference between dietary variables. These data confirmed the anthropometric measurement results (the correlation between MR muscle area and anthropometric measurement calculated muscle area was *r* = 0.88). There was no significant change in fat mass, while muscle mass (FFM) showed a significant increase (*p* = 0.0003), as reported in Table 1. No significant differences were detectable between diet groups.

### 3.3. Muscle Fiber Type Composition and Muscle Fiber Mechanics

**Figure 3:**
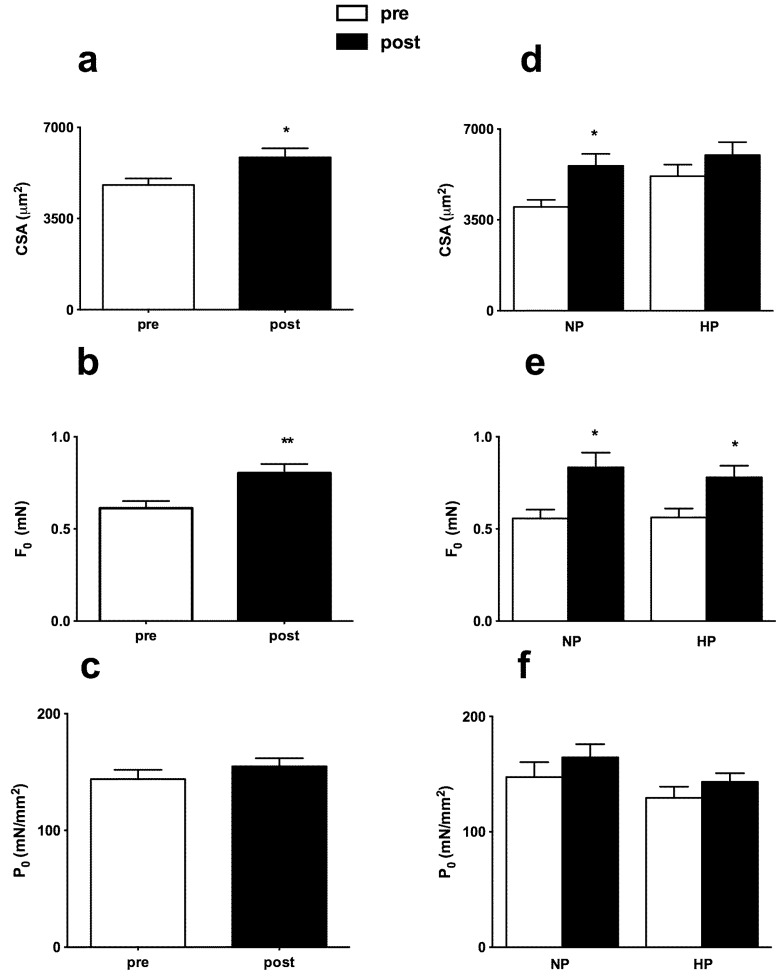
Effects of RT and protein supply (NP and HP) on Cross-Sectional Area (CSA), Isometric Force (Fo), and Isometric tension (Po). Merged NP + HP data are shown in (**a**–**c**), while individual data for the NP and HP groups are shown in (**d**–**f**). A paired Student’s *t*-test was used for merged data (**a**,**d**), while mixed-model ANOVA (with one between-subjects factor and one within-subjects factor) was used for HP and NP analysis (**b**,**c**,**e**,**f**). Means and Standard Errors. * *p* < 0.05; ** *p* < 0.005 (pre- *vs.* post-).

The impact of RT protocol on LDM was detectable at a cellular level. Single muscle fibers dissected from LDM showed a significant increase of average CSA and of average isometric force (Fo) after eight weeks of training, while the isometric specific tension (Po) was unchanged (see Figure 3a–c, where the supplementation and non-supplementation groups are pooled together to better show the effect of training).

When the effects of resistance training (pre *vs.* post) and supplementation (HP *vs.* NP) were separated (see Figure 3d–f), ANOVA showed a significant effect of training but no effect of protein supplementation on Fo, while Po showed no significant changes. Interestingly, the increase of CSA reached statistical significance only in NP (main effect for diet: interaction *p* = 0.835; main effect: *p* = 0.006) (Figure 3d).

**Figure 4:**
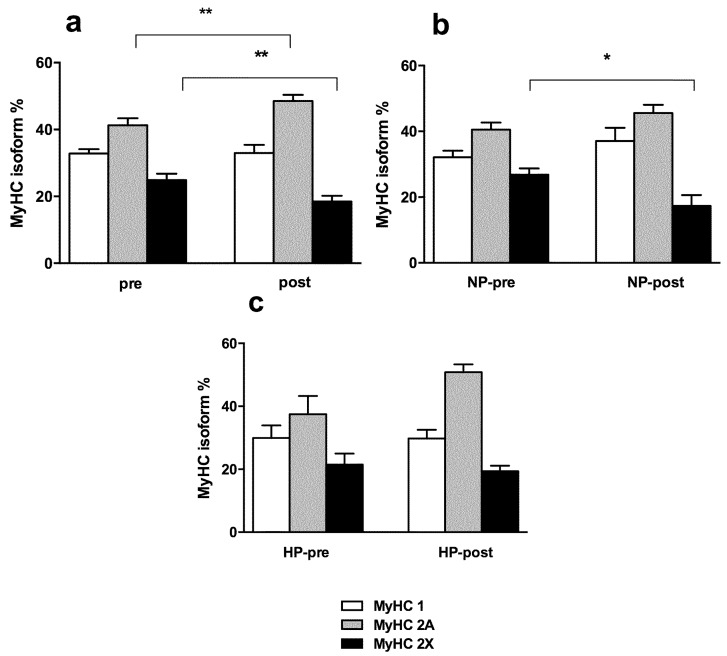
MyHC isoform distribution in biopsy samples collected pre- and post-training. (**a**) All subjects pooled together (*n* = 18); (**b**) only subjects with lower protein intake (NP, *n* = 9); (**c**) only subjects with higher protein intake (HP, *n* = 9). Means and Standard Errors. * *p* < 0.05, ** *p* < 0.005.

Electrophoretic analysis of MyHC isoform distribution in biopsy samples showed minor but significant changes after RT and in relation to protein intake (Figure 4). When considering merged NP and HP (Figure 4a), training induced an increase in MyHC 2A percentage, while the percentage of MyHC 2X decreased after eight weeks of training. ANOVA showed that the nutritional regimen influenced the MyHC isoform profile only in the NP group, but not HP group (time × diet interaction significant effect, *p* = 0.039 with a simple main effect for diet: *p* = 0.0229), a significant decrease of MyHC 2X proportion occurred (see Figure 4b,c). The percentage of MyHC 1 or slow fibers remained constant. The analysis of the distribution of fibers classified according to their MyHC isoform composition showed that the proportion of pure slow (type 1) and pure fast 2A fibers was not modified by training or diet, while hybrid fibers (1/2A and 2A/2X) significantly increased after the training period (data not shown) (interaction *p* = 0.75; main effect of time: *p* = 0.0156). It is worth noting that pure 2X fibers, *i.e.*, fibers expressing only MyHC 2X, were a minority and not sufficient for statistical analysis.

**Figure 5:**
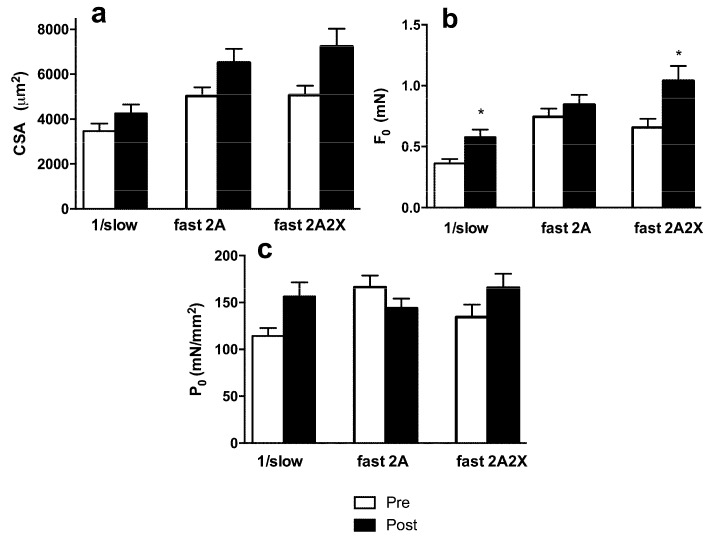
Response of individual fiber types, classified on the basis of their MyHC isoform composition, to RT training. Fibers from NP and HP subjects are pooled together. Means and Standard Errors. Significant difference between post- and pre-training: * *p* < 0.05.

**Figure 6:**
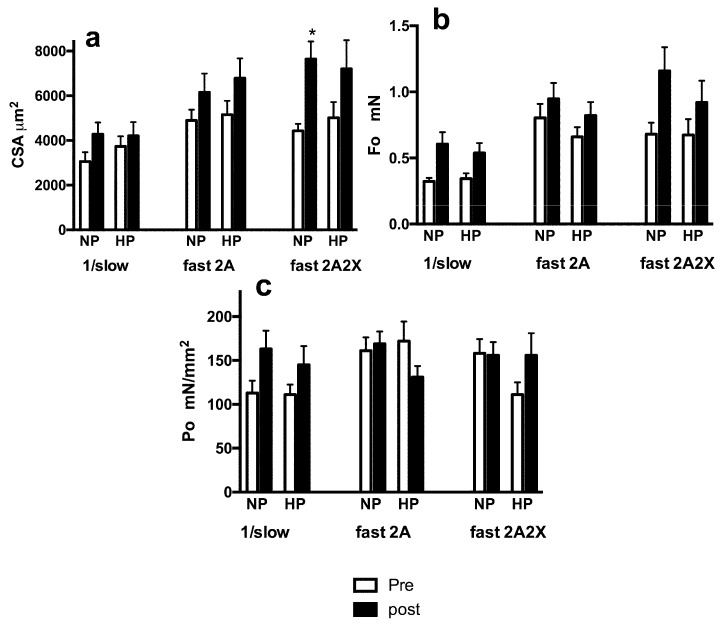
Response of individual fiber types, classified on the basis of their MyHC isoform composition and treatment (HP *vs.* NP) to RT training. Means and Standard Errors. Significant difference between post- and pre-training * *p* < 0.05.

The effects of training on the thickness (CSA) and contractile parameters (Fo, Po) of single fibers classified on the basis of the MyHC isoform expressed are reported in Figure 5. The analysis is restricted to the three more abundant groups, *i.e.*, pure slow or type 1 fibers, pure fast 2A fibers, and hybrid 2A/2X fibers. Pure 2X fibers were not considered, being a rather small group (see above). As can be seen, all fiber types showed hypertrophic response, as indicated by the increase in CSA (Figure 5a), while only 2A/2X NP fibers showed a significant improvement (see Figure 6a). No changes in specific tension were detected (Figure 5c), while an increase in Fo was observed in slow and 2A2X fibers (see Figure 5b). When single fibers classified on the basis of the MyHC are analyzed by treatment group (HP and NP), NP showed a significant, albeit at the limit of statistical significance, greater increase in 2A2X fibers’ Fo (*p* < 0.05) (Figure 6b).

**Figure 7:**
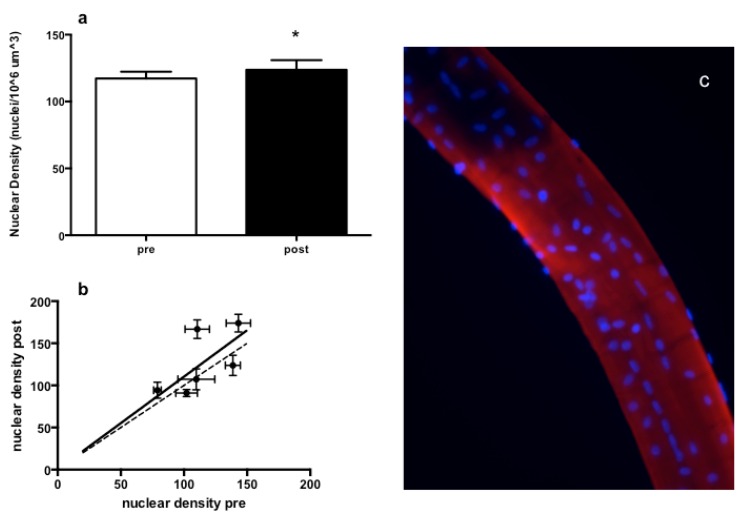
Increase in nuclear density after eight weeks of RT. * *p* < 0.05. (**a**) Average values pre- and post-training from six subjects each with eight fibers analyzed in each condition; (**b**) correlation between the average myonuclei density post- (ordinate) and average density pre- (abscissa) training calculated for each subject. Two subjects showed no change or a slight decrease, while four subjects showed an increase. The regression line is Y = 1.10 ± 0.08X, not significantly different from the perfect line with slope 1; (**c**) Single muscle fibers stained with anti-actinin antibody (red) and with Hoechst nuclear staining (blue).

In a set of fibers from six randomly selected subjects (three HP and three NP), myonuclear density (Figure 7c) was calculated as described in the Methods section. As shown in Figure 7a, a significant increase in myonuclei density was found after eight weeks of RT training, indicating that the average size of the myonuclear domain decreases by approximately 9% from 9851 to 8356 µm^3^. If the six subjects are analyzed separately, the increase is present in four and virtually absent in two subjects (see Figure 7b). Moreover, there was no significant correlation between nuclear density and muscle fiber type or between nuclear density and CSA.

## 4. Discussion

A major goal of our research was to investigate the effect of a high protein diet on LDM response to RT. The present study shows that eight weeks of RT were sufficient to increase upper limb muscle size and strength as well as LDM muscle fibers CSA and isometric force without any detectable differences between normal protein and high protein groups. It is the first time that the adaptive response to resistance training (RT) and protein supplementation has been studied in LDM fibers. LDM is not only the largest muscle in the human body, but is also an important target for training in view of its relevance in many athletic gestures. As stated in the introduction, human upper limb muscles represent an ideal target to study the effects of specific interventions based on RT and diet, being free of the postural and locomotion tasks in which lower limb muscles are involved for many hours every day.

The vastus lateralis has been, for both muscle disease diagnosis and for muscle and exercise physiology, the most commonly used muscle for sampling. The choice has been based on the possibility of accessing the fibers without risk of damaging important nerves and blood vessels to the bulk of a muscle involved in many types of exercise from cycling and running to leg extension (see [29]). Biopsy sampling of upper limb muscles, despite their distinct tasks in posture and locomotion and their specific involvement in some muscle diseases (for example, FSHD or FacioScapuloHumeral muscular Dystrophy [35] and Limb Girdle dystrophies [36]), has been performed much less often. Besides our recent paper [17], there are only two published papers [37,38] describing biopsy sampling of LDM, and, in both cases, the sampling was based on surgical open biopsies. In our studies, a fine needle biopsy was adopted to minimize pain and discomfort to participants and we focused on the adaptive response of single muscle fibers to RT and high or normal protein diet. 

As expected, eight weeks of RT increased strength performance (lat pulldown), reflecting the increase in muscle area of related muscle groups (upper limb), as shown by MR and the increases of body muscle mass and in a reasonable measure, the improvement in neuromuscular activation [39]. On the other hand, this kind of RT protocol did not show any significant effect on body fat. At a cellular level, the results obtained showed that eight weeks of RT elicited a significant hypertrophic response of the LDM (increase of CSA) in all fiber types (+22%) with a parallel increase in Fo and no change in Po. The latter findings are in agreement with previous findings from other studies [40,41] but in contrast with those of Pansarasa [42], who found an increase in specific force (Fo/CSA). This discrepancy may be due to different characteristics of subjects and a different duration of training: one year in Pansarasa’s study *versus* eight weeks in our study. Interestingly, the most responsive fibers were hybrid fast 2A/2X fibers with an increase in CSA of 61% and in force of 60.2%. It is worth noting that LDM seems to have a relatively high percentage of 2X and 2A/2Xfibers (merged) *i.e.*, 32.3% [17]. Interestingly, LDM fibers showed the same shift in myosin isoform in response to RT (a shift from 2X to 2A, which corresponds to an increase of mixed fibers) as reported for the quadriceps vastus lateralis [43,44] (see Figure 6). The change in nuclear density deserves some comments: the increase in nuclear density indicates that the number of nuclei is increased more than the volume of the fiber. This indicates that RT produces an effective activation of satellite cells or of other stem cells that may play the role of nuclear donor for the fibers.

In regard to the effects of different intakes of protein, our results confirm previous research carried out by Verdijk and co-workers in an elderly population [45] that demonstrated the inconsistency of protein supplementation on muscle fiber hypertrophy. They are also in agreement with the results of a recent paper by Boone and collaborators [46] on untrained men. However, these are in contrast with other studies [11,47,48,49] that showed a greater muscle hypertrophy with higher protein intake. The ability of amino acids to regulate protein synthesis in muscle is believed to be mediated through the mammalian target of rapamycin (mTOR) as part of a pathway involving downstream targets [50]. Nevertheless, conclusions based on the data available in the literature are not so straightforward. For example, in young men, protein and/or essential amino acids supplementation in combination with resistance training has been shown to significantly increase myofiber CSA more than a non-energetic or carbohydrate placebo [47,51]. Other studies have demonstrated that protein and/or essential amino acids are more effective than carbohydrate or a placebo to increase muscle mass and muscle CSA [48,52]. Conversely, other studies failed to demonstrate that protein and/or amino acid ingestion has a significant effect on myofiber size or muscle body mass during resistance training [45,53]. In our study, the lack of effects of protein supplementation on skeletal muscle hypertrophy may be due to the specific muscle characteristics or, more likely, to the different weight lifting experience. Our subjects are recreationally active sport science students and it could be hypothesized that they can reach lower exercise intensity compared to more experienced athletes and that such intensity represents a key factor for muscle fiber damage and subsequently for a protein-dependent effect. It is, however, important to underline that, when considering the two nutritional protocols, our data evidenced that protein supplementation blunts the well-known tendency to reduction of MyHC 2X expression during resistance training [54]. The decrease in MyHC 2X expression was not present in the HP group (see Figure 6c), suggesting that a higher protein uptake could interfere with muscle fiber type shift. The mechanism underlying this effect is not yet completely understood, but it is worth underlining that the available data show that a reduced protein intake without any caloric restriction induces a shift from 2X to 2A [55] and that an increased amino acid supply in trained rodents on a treadmill triggered a shift towards 2B fibers [56]. In rodents, 2B fibers play a functional role similar to the 2X fibers in humans, being the fastest and most powerful group of muscle fibers (see Bottinelli and Reggiani [57]).

In conclusion, this study provides novel and potentially useful information about latissimus dorsi muscle fibers’ response to RT and a normal or high protein diet and, conceivably, about the eventual development of new, efficient training programs for athletes but also for patients who must recover LDM function. Our data give evidence that the adaptive response to RT at the single fiber level in a large upper limb muscle shows a substantial similarity to that reported in lower limb muscles. Our findings show the hypertrophic response of the muscle fibers, with proportional change in force generation and, moreover, demonstrate the typical shift (observed in the vastus lateralis) in myosin isoform from 2X to 2A with an increase in hybrid fibers also in LDM. The results concerning the effects of increased protein intake point to a more specific effect that does not end in a change in muscle fiber size, but involves the myosin isoform transition. Our data show the blunting effects of protein supplementation on the myosin 2X isoform shift. Muscle fibers expressing myosin 2X are the fastest and most powerful in the human body, and their preservation is one goal of high-level contractile performance.

## Figures and Tables

**Table 1:** Sample diet composition of two subjects with similar calculated daily caloric needs. The calculated individual needs are, respectively 2200 kcal/day for the HP subject and 2350 for the NP subject. The values shown in this table were based on analysis of the food diaries.

Total Daily Calories Kcal	HP	NP
	**2226.5 ± 60.2**	**2207.5 ± 41.7**
CHO g	261.6 ± 7.1	333.7 ± 6.3
FAT g	61.8 ± 1.7	61.3 ± 1.2
PRO g	155.9 ± 4.2	80.1 ± 6.2
CHO kcal	1046.4 ± 28.3	1335.1 ± 25.1
FAT kcal	556.6 ± 15	551.7 ± 10.7
PRO kcal	623.4 ± 16.7	320.2 ± 25.1
CHO %	47 ± 2	62 ± 3
FAT %	25 ± 1	25 ± 2
PRO %	28 ± 1	13 ± 1

**Table 2:** Anthropometric variables before and after eight weeks of resistance training (all subjects). Mean ± Standard Error are reported. (FM = fat mass; MM = muscle mass; MA = muscle area); upper limb was the dominant one.

Variable	Pre	Post	Significance
Total body FM Kg	12.46 ± 5.62	12.54 ± 4.47	n.s.
Total body MM Kg	33.77 ± 6.23	35.5 ± 6.67	*p* = 0.0003
Upper limb MA cm^2^	45.13 ± 3.3	47.94 ± 4.4	*p* = 0.024
